# Perceived clinical challenges when treating patients from different cultures: A study among psychiatry trainees in Norway

**DOI:** 10.1177/13634615241296297

**Published:** 2024-12-24

**Authors:** Morten Sandbu, Anne Cecilie Javo, Suraj Bahadur Thapa, Karin Isaksson Rø, Valjbona Preljevic, Reidar Tyssen

**Affiliations:** 1 Institute of Basic Medical Sciences, 6305 University of Oslo; 2 155272 Oslo University Hospital; 3 Sámi National Competence Center for Mental Health (SANKS); 4 Institute of Clinical Medicine, 6305 University of Oslo; 5 Institute for Studies of the Medical Profession; 6 Specialist Practice in Psychiatry in Oslo

**Keywords:** psychiatry, clinical challenges, foreign and native doctors

## Abstract

The growing number of migrant patients in western countries calls for better cross-cultural competence among health providers. As workplaces, hospitals have become increasingly multicultural, and many doctors are themselves of foreign origin, including psychiatrists. The aims of this study were to explore what clinical challenges International Medical Graduates (IMGs) and native-born Norwegian doctors training in psychiatry perceived when treating patients from other cultures, and what factors might be associated with such cross-cultural challenges. We developed a six-item inventory of perceived cross-cultural clinical challenges (PCC), to assess what cross-cultural problems trainees in psychiatry found most challenging. The PCC was completed by 216 trainees who also reported on individual- and work-related background factors. Comparisons of PCC between the two groups were done by one-way analysis of variance, and associations between PCC and background factors were analyzed by linear multiple regression. The overall response rate was 93%. Native -born Norwegian doctors reported higher levels of PCC than did IMGs. Both native-born Norwegian doctors and IMGs rated “assessing psychosis,” “assessing suicide risk,” and “lacking tools in cross-cultural consultations” as the most demanding challenges in cross-cultural consultations. Independent factors associated with higher PCC included being a native-born Norwegian doctor and experiencing high levels of work–home conflict. The findings suggest that trainees in psychiatry may need more training and better tools in cross-cultural assessment of mental disorders. Possible differences in PCC between native-born doctors and IMGs should be taken into consideration when developing mentoring programs, as should the doctors’ work–home conflict level, which might impact the PCC.

## Introduction

Because of greater globalization, an increasing number of doctors in specialist training are migrating to countries with a linguistic and cultural background different from their own, with psychiatry being no exception (Kirmayer et al., 2018). In Norway, psychiatry is the medical discipline with the highest proportion (24%) of practicing International Medical Graduates (IMGs). Likewise, a higher number of patients are themselves migrants. In the host country, they will most often be treated by doctors who belong to a different culture. This leads to a situation in which language and cultural barriers may frequently arise in clinical practice (Hall et al., 2004; Mahajan & Stark, 2007; Memon et al., 2016a, 2016b; Sockalingam, Hawa et al., 2014; Sockalingam, Khan et al., 2014). Obviously, language barriers are important in cross-cultural encounters and may influence performance and clinical skills, but cultural barriers might be as important (Michalski et al., 2017). Culture has been defined as the system of knowledge, concepts, rules, and practices that are learned and transmitted across generations, but culture is still open, dynamic, and undergoes continuous change over time (American Psychiatric Association, 2013).

However, there is a lack of empirical studies on what specific clinical challenges doctors face when they diagnose and treat patients from a culture other than their own (Ekblad & Kastrup, 2013). Although studies have described specific psychiatric symptoms, such as depression, dissociation, and hallucinations in a cross-cultural perspective (Fung et al., 2020; Laroi et al., 2018; Lehti et al., 2009), others have focused on the need for more cross-cultural training of professionals in the psychiatric field (Kirmayer et al., 2018). Few studies have examined what clinical challenges and needs of specific skills doctors in psychiatry are actually experiencing in their cross-cultural consultations. Also, we do not know whether there are differences in PCC between IMGs and native doctors. Previous research on IMGs in western countries has primarily focused on the need for overcoming integration barriers, professional isolation, cultural differences in communication style, gender roles, and difficulties related to language (Hall et al., 2004; Launer, 2018; Michalski et al., 2017; Sturesson et al., 2019).

In 2013, The American Psychiatric Association included the Culture Formulation Interview (CFI) in the Diagnostic and Statistical Manual of Mental Disorders (DSM-5) diagnostic system (American Psychiatric Association, 2013). The CFI intended to support culturally informed categorization of mental disorders and to respond to the cultural backgrounds and social contexts of patients (Strand & Baarnhielm, 2022). Although training programs on DSM-5 CFI may improve general cultural competence in psychiatry residents (Mills et al., 2017), use of the CFI may not fully meet the trainee's specific needs related to cross-cultural clinical skills in psychiatric assessments of patients. However, to our knowledge, there are no studies on doctors’ perceived challenges with respect to clinical skills in cross-cultural psychiatry.

The ultimate goal of our current research project was to inform a future voluntary mentoring group intervention about transcultural clinical challenges for doctors training in psychiatry. Hence, we needed to tailor the curriculum to the perceived needs of the trainees. Because we lacked knowledge about their needs, we intended to identify the most important cross-cultural clinical challenges experienced by both IMGs and native-born Norwegian trainees. This was done in a two-step design. First, we developed an inventory to measure cross-cultural clinical challenges by asking selected trainees in psychiatry as well as experienced specialists in transcultural psychiatry to list presumed clinical challenges when assessing patients from another culture. The suggested items were then used to construct an inventory to measure such challenges (see the section “Measures” for more detailed information about the psychometric procedures). Subsequently, we performed the current study, using the inventory as a tool.

Based on previous studies on variables associated with doctors’ difficulties in their general clinical work (Lai & Plakiotis, 2020; Rotstein & Jenkins, 2017; Røvik et al., 2007; Tyssen et al., 2000), we hypothesized that work-related factors might also impact the doctors’ perception of difficulties in cross-cultural consultations. Factors that we wanted to examine were stage of career in psychiatry training, former experience of cross-cultural consultations, support from colleagues, workload (number of working hours), and perceived stress in the workplace. We also included personality trait (neuroticism) as a factor to control for negative affectivity and to increase the validity of self-reported findings about work-related factors (Depue & Monroe, 1986).

Against this background, we explored the following research questions:

1. What clinical challenges do doctors training in psychiatry in Norway perceive when they treat patients from other cultures?
2. Are there differences between IMGs and native-born Norwegian doctors in their perception of cross-cultural clinical challenges?
3. What individual and work-related factors are associated with doctors perceived clinical challenges in cross-cultural consultations?

## Methods

### Subjects and procedure

Study participants were doctors training in psychiatry, including both native-born Norwegian doctors and IMGs. An IMG was defined as a doctor who had a medical education from abroad and a first language (mother tongue) other than Norwegian. Participants were recruited at the five national mandatory specialist courses held at different locations in Norway during 2019. This was considered an optimal recruiting method because all psychiatric trainees in Norway have four mandatory week-long courses during a two-year period of their education—one course every six months. With respect to professional orientation, all trainees in psychiatry in Norway were receiving two-year mandatory psychodynamic psychotherapy supervision at the time of participation.

All participants were asked to complete an inventory regarding perceived clinical challenges in cross-cultural consultations. In addition, they were asked about individual and work-related factors that could impact clinical work with patients from other cultures. The distribution and collection of questionnaires at the respective sites was done by two of the authors (MS and RT), both of whom are psychiatrists. While completing the forms, the participants had the opportunity to ask questions to clarify possible misunderstandings. The same authors (MS and RT) answered these queries.

### Ethics

The authors asserted that all procedures contributing to this work complied with the ethical standards of the relevant national and institutional committees on human experimentation and with the Helsinki Declaration of 1975, as revised in 2008. All procedures were done according to the guidelines of the regional ethical committee (REC Sør-Øst C, approval number 48765, dated 28 November 2019). All participants were informed about the study both verbally and in writing. Participation was voluntary and completion of the questionnaire was regarded as giving written consent. The participants were always free to interrupt or end their participation.

### Measures

#### Perceived cross-cultural clinical challenges

We used conventional psychometric procedures to construct this inventory (Bowling, 2023). Initially, we (a) asked a panel of IMGs and native-born Norwegian psychiatric trainees, as well as a focus group of both native and foreign experts in transcultural psychiatry, the following question: “What clinical challenges and assets do you experience when you meet a patient with a lingual or cultural background different from your own?” On face validity, 24 items emerged from these initial inquiries (Appendix 1 available online). Subsequently, we (b) invited 48 trainees to score these items, in a psychometric validation to develop a shorter instrument. These items were scored on a 7-point Likert scale (1, incorrect to 7, fits very well) The 48 trainees (including both IMGs and native-born Norwegian doctors) were recruited from the two largest psychiatric hospitals in the Oslo capital region. Principal component analysis (PCA) with varimax rotation and a scree plot was used to analyze the data, and this confirmed the concurrent validity of one factor. A scale reduction (reliability analysis) condensed the original 24 items to only 6. This six-item scale yielded an index of perceived cross-cultural clinical challenges (PCC) that was used as the principal variable in the current study.

Three of the six items pertain to specific psychiatric assessment skills that are frequently needed in psychiatric practice, such as assessing psychosis, suicide risk, and risk of violence. Other reported challenges were difficulties in making appropriate treatment plans for patients of other cultural backgrounds, lack of appropriate tools to assist in diagnostic assessments of patients from other cultures, and insufficiency in providing adequate services to cultural minorities. The sum of item scores was used as a measure of PCC. Cronbach's alpha values for the PCC in the initial phase and for the current study sample were.80 and.85, respectively.

#### Associated factors (independent variables)

In addition to age (measured as a continuous variable) and gender (female coded 1, male coded 2), we included the following variables in the regression analysis of PCC:

Cultural background. The cultural background of the participants was scored as native-born Norwegian = 1 and IMG = 0.
Stage of specialist training. To define the participants’ stage of training, the mandatory training course of which they attended was used as an indicator. The courses were marked I, II, III and IV, indicating the sequential training stage, and the variable was dichotomized. Courses I and II were coded 1, and courses III and IV were coded 2.
Frequency of cross-cultural consultations. The question: “How often do you meet patients from a culture different from your own?” was categorized as: “daily”, “weekly”, “monthly” or “yearly”. This item was dichotomized as “daily” (1) and “weekly or more seldom” (0)*.*
Support from colleagues. This item included the following two questions: “To what degree do you enjoy working with your colleagues?” and: “To what degree do you feel support from your colleagues?”. The responses were scored on a 7-point Likert scale, from 1 (not at all) to 7 (to a very high degree). The scores from the two items were summed for a total score of perceived support. This variable has previously been validated in Norwegian longitudinal studies of job stress and perceived mastery of clinical work among established doctors in Norway (Belfrage et al., 2018; Røvik et al., 2007).
Work stress-related factors. These were derived from a modified Norwegian version of Cooper's Job Stress Questionnaire (Røvik et al., 2007; Sutherland & Cooper, 1992; Tyssen et al., 2000). The questionnaire included 23 items headed by the question: “To what degree do the following situations make you feel stressed?” All items were scored on a scale from 1 (not at all) to 5 (very much). A PCA and scale reduction for our sample identified the following three factors: emotionally demanding patient work (eight items), work–home conflict (six items) and demanding role as a physician (four items).
Emotionally demanding patient work included items such as: “Dealing with problem-patients,” “Twenty-four-hour responsibility for the patients’ lives,” and “Worrying about patients’ complaints”. Cronbach's alpha for the eight items in our sample was. 78.
Work–home conflict was assessed by items such as: “Demands of your job on family life” and “Balancing oneself between work and private life.” Cronbach's alpha for the six items in our sample was.83.
Demanding role as a physician was measured by three items such as: “Dealing with friends as patients” and “Dealing with relatives as patients.” Cronbach's alpha was. 82.
Number of working hours per week was measured as a continuous variable.
Neuroticism trait. Questions about neuroticism were taken from the Basic Character Inventory (Torgersen, 1980; Tyssen et al., 2000). The nine-item inventory resembles Eysenck's classical neuroticism scale with items about low self-esteem and self-criticism. The responses were made dichotomous—1 (agree), 0 (do not agree)—and a sum score from 0 (low) to 9 (high) was computed.

Descriptions of these variables are presented in Table 1.

**Table 1.** Description of the independent variables.

Independent variable	Range	Mean (*SD*)	%
		
Age	(25–63)	35.4	(61)
Female gender		68	
Native-born Norwegian doctor		83	
Stage of training (advanced)		32	
Frequency of cross-cultural consultations (daily)		22	
Colleague support	(6–24)	11.21	(2.9)
Emotionally demanding patient work	(12–37)	23.50	(5.3)
Work–home conflict	(6–30)	16.58	(4.7)
Demanding role as a physician	(4–20)	8.30	(3.9)
Numbers of working hours per week	(25–80)	43.50	(6.3)
Neuroticism trait	(0–9)	3.73	(2.3)

### Statistical analyses

To explore which specific skills the two groups of doctors found the most challenging, and to assess the difference between the mean scores, we conducted Student's *t*-tests. To measure the relative magnitude of the differences between the means, i.e., the effect size, we calculated Cohen's *d*; values of 0.2–0.5 indicate a small effect size, 0.5–0.8 a medium effect size, and >0.8 a large effect size.

Bivariate linear regression analysis was used to test the associations between each of the predictor variables and the dependent variable. To explore the relative importance of the predictor variables, we computed a block-wise hierarchical multiple regression analysis with PCC as the dependent variable and entered the predictor variables in three blocks (forced entry). This yielded three models of multivariate analysis. The contribution of each block was expressed by differences in explained variance (adjusted *R*^2^). The level of significance chosen was 5%, with 95% confidence intervals (CI).

## Results

The response rate in this study was 93% (216/233). Of the respondents, 17% (37/216) were IMGs and 83% (179/216) native-born Norwegian doctors. Women constituted 68% of the sample: 70% among the IMGs and 68% among the native-born Norwegian doctors.

### Levels of perceived cross-cultural clinical challenges

The mean level of PCC among all psychiatry trainees was 27.6 (*SD* = 6.8): 27.5 (6.6) among the female trainees and 26.9 (7.3) among the male trainees. There was no significant gender difference. Native-born Norwegian doctors reported higher levels of PCC than did IMGs: 28.8 (*SD* = 6.2) vs 23.8 (7.2), *p *< .001, Cohen's *d *= 0.73.

As seen in Table 2, the most demanding clinical challenges reported by both native-born Norwegian doctors and IMGs were: “Assessment of psychosis” and “Lacking tools in cross-cultural consultations.”

**Table 2.** Perceived cross-cultural clinical challenges.

Native-born Norwegian doctors			International Medical Graduates		
	Mean	*SD*	Mean	*SD*	*p*-value; Cohen's *d*
Perceived cross-cultural clinical challenges items^a^					
Assessment of psychosis	5.50	1.40	4.64	1.64	.001; 0.56
Lacking tools in cross-cultural consultations	4.84	1.50	3.97	1.81	.010; 0.52
Assessment of suicide risk	4.79	1.33	3.69	1.72	.001; 0.72
Assessment of violence risk	4.58	1.43	3.72	1.61	.002; 0.52
Development of individual treatment plans	4.31	1.43	3.64	1.82	.042; 0.41
Psychiatric service is not relevant and sufficient for cultural minorities	3.24	1.23	3.86	1.71	.045; 0.42

^a^ The items were scored on a 7-point Likert scale, from 1 (do not agree) to 7 (agree very much).

The largest mean differences between native-born Norwegian doctors and IMGs were on the items “Assessment of suicide risk” (4.79 vs 3.69; *p *= .001, *d *= 0.72), “Assessment of psychosis” (5.50 and 4.64; *p *= .001, *d *= 0.56), “Assessment of violence risk” (4.58 vs 3.72, *p *= .002, *d *= 0.52), and “Lacking tools in cross-cultural consultations” (4.84 vs 3.97, *p *= .010, *d *= 0.52).

### Factors associated with perceived cross-cultural clinical challenges

#### Bivariate analysis

Table 3 shows that the significant associated factors of PCC were native-born Norwegian doctor, frequent transcultural consultations, emotionally demanding patient work, high work–home conflict, and high levels of neuroticism.

**Table 3.** Predictors of ‘perceived cross-cultural clinical challenges’ among trainees in psychiatry.

Variables entered in the analyses	Bivariate analyses		Model 1 (adjusted *R*^2 ^= 0.08)		Model 2 (adjusted *R*^2 ^= 0.17)		Model 3 (adjusted *R*^2 ^= 0.18)	
	Crude beta	95% CI	Adjusted beta	95% CI	Adjusted beta	95% CI	Adjusted beta	95% CI
Age	−0.11	−0.25 to 0.04	−0.06	−0.21 to 0.09	−0.03	−0.18 to 0.12	−0.04	−0.18 to 0.11
Female gender	−0.99	−2.93 to 0.94	−0.99	−2.91 to 0.94	0.08	−1.89 to 2.05	0.32	−1.67 to 2.30
Native-born Norwegian doctor	5.06***	2.71 to 7.41	4.96***	2.56 to 7.35	4.09**	1.32 to 6.86	3.89**	1.12 to 6.66
Stage of training	1.55	−0.39 to 3.48			0.47	−1.46 to 2.40	0.49	−1.43 to 2.41
Frequency of cross-cultural consultations	2.98*	0.83 to 5.13			−1.32	−3.85 to 1.21	−1.41	−3.93 to 1.12
Colleague support	0.16	−0.24 to 0.55			0.07	−0.27 to 0.40	0.01	−0.36 to 0.34
Emotionally demanding patient work	0.26**	0.09 to 0.43			0.13	−0.06 to 0.33	0.08	−0.12 to 0.29
Work–home conflict	0.34***	0.16 to 0.53			0.28*	0.05 to 0.52	0.29*	0.05 to 0.52
Demanding role as a physician	0.11	−0.3 to 0.34			0.06	−0.23 to 0.24	0.01	−0.22 to 0.25
Number of working hours per week	−0.18	−0.28 to 0.01			−0.12	−0.26 to 0.03	−0.11	−0.25 to 0.03
Neuroticism trait	0.61**	0.22 to 1.0					0.35	−0.09 to 0.79

*p < .05; **p < .01; ***p < .001.

#### Multiple regression analysis

In the first model (Table 3), we entered age, gender, and native-born Norwegian doctor/IMG as predictor variables. Only “being a native-born Norwegian doctor” (beta = 4.96, 95 CI 2.56 to 7.35, *p *< .001) remained significant in the multiple regression analysis. The adjusted *R*^2^ of this model was 0.08, indicating that these variables explained 8% of the variance in PCC.

In the second model, we entered work-related variables, i.e., frequency of transcultural consultations, support from colleagues, emotionally demanding patient work, work–home conflict, and number of working hours per week as predictor variables. Being a native-born Norwegian doctor (beta = 4.09) and work–home conflict (beta = 0.28) were the only variables that remained significant when adjusted for the other variables. The *R*^2^ of model 2 was 0.17, which means that the entry of these variables into the model contributed an additional 9% of the variance.

In the third model, we entered neuroticism as the last predictor variable, but this variable was not significant when controlled for all other variables, whereas being a native-born Norwegian doctor (beta = 3.89) and work–home conflict (beta = 0.29) remained significant. The explained variance of the third (final) model was 18% (*R*^2 ^= 0.18).

We also examined whether there were any interaction effects between the significant adjusted predictors and gender, as well as being a native-born Norwegian doctor/IMG. No significant interactions emerged. This means that the effects of the significant predictors were similar for both genders, and that the effect of work–home conflict was similar for both native-born and foreign doctors.

## Discussion

To our knowledge, this is the first empirical study on PCC among native-born doctors and IMGs training in psychiatry in a host country. Both groups reported that “assessing psychosis” and “lacking tools in cross-cultural consultations” were their most prominent challenges. The native-born Norwegian doctors experienced higher levels of cross-cultural challenges in their clinical work than did the IMGs, in particular with respect to assessing suicide risk. Independent factors associated with cross-cultural clinical challenges were native-born Norwegian doctor and experiencing higher levels of work–home conflict.

### Perceived cross-cultural clinical challenges

One of the cross-cultural clinical challenges that both native-born Norwegian doctors and IMGs training in psychiatry found most difficult was assessing psychosis. This is not surprising. To assess psychosis, language competence as well as knowledge about culture-bound syndromes and culturally based ideas and health beliefs are all required (Fung et al., 2020; Memon et al., 2016a, 2016b; Şar, 2021). A culturally sensitive understanding of the patient is also a necessary competency for the therapist. This is all paramount to understand what is reality or not in the patient's perceptions (Laroi et al., 2018). Since the 1950s, research in medical anthropology and sociology has documented how culture influences the boundary between normal and abnormal behavior, the patterning of symptoms, how patients narrate their symptoms, and the way clinicians interpret symptoms into psychiatric diagnoses (Lewis-Fernández et al., 2020). Further, culture affects patients’ explanatory models as well as their help-seeking patterns (Lewis-Fernández et al., 2020). Hence, cross-cultural assessment of psychosis is complicated, indeed.

Further, the assessment of suicide risk was reported as an important challenge in cross-cultural consultations. Evaluating such risk is generally found to be one of the most challenging tasks in psychiatric work (Simon et al., 2018). This is partly due to the problem of extrapolating risk factors from a statistical group level to an individual level. Completed suicide is a quite rare event, with a complex nature (Turecki & Brent, 2015), and is perceived as challenging because of the serious and possibly fatal consequences of misjudgment. To evaluate suicide risk in a cross-cultural context is particularly complicated because cultural attitudes to suicidal behavior differ cross-culturally, including religious beliefs’ ability to protect a person from committing suicide (Lew et al., 2021; Lizardi & Gearing, 2010; Rosmarin et al., 2013). Patients’ methods of committing suicide may differ across cultural and social contexts, partly because of different access to tools, such as firearms and self-poisoning agents like medicines and pesticides/insecticides. This makes suicide risk assessment of patients from other cultures even more difficult for doctors (Bachmann, 2018; Cruzeiro Szortyka et al., 2021; Stack & Kposowa, 2008).

Lack of tools in cross-cultural assessments was the second highest rated challenge by both groups of doctors. More studies are warranted as to what kind of tools might be helpful. The CFI in the DSM-5 has been used in several countries to enhance assessment of patients with a different culture (Kirmayer et al., 2016), and use of the CFI might lead to major revisions of clinical psychiatric diagnoses (Aggarwal et al., 2020; Bäärnhielm et al., 2014; Rosso & Bäärnhielm, 2012). In a meta-analysis of 25 peer-reviewed studies, patients and clinicians reported that using the CFI improved clinical rapport (Aggarwal et al., 2020). This also emphasizes that the CFI helps to disclose the patient perspective and is of major importance when assessing the needs and aims of psychotherapy. However, the CFI is not frequently used by many psychiatrists, including in Norway, in spite of its obvious benefits (Ramírez Stege & Yarris, 2017; Rosso & Bäärnhielm, 2012). The CFI may be regarded as too time-consuming to use during hectic workdays. Although doctors training in psychiatry in Norway are all educated in use of the CFI, our study suggests that they may need additional tools, especially when it comes to cross-cultural assessments of psychosis, suicide risk, and violence risk. The perceived challenges presented in this study may suggest which clinical topics and skills to focus on when developing local training and support groups for trainees in psychiatry.

Our study suggests that the doctors perceived insufficient competency in cross-cultural assessment, because they rated assessing psychosis, suicide risk, and violence risk as being particularly challenging. Hopefully, future research might increase our knowledge about how to improve assessment tools that doctors in psychiatry find helpful in their cross-cultural clinical assessments. The development and use of such tools is important, because it might enhance cultural awareness within the mental health services (Islam et al., 2015).

### Differences between IMGs and native Norwegian doctors in PCC

The IMGs reported lower levels of PCC than did the native-born Norwegian doctors, even when other variables were controlled for. This finding is in accordance with a previous Norwegian qualitative study showing that IMGs in psychiatry experienced fewer cultural problems in their encounters with patients than did native-born Norwegian doctors (Sandbu et al., 2015). By contrast, the native-born Norwegian doctors in that former study experienced that their IMG colleagues could misunderstand Norwegian culture, interpret clinical situations differently, and that their medical assessments might differ from their own.

One explanation of the finding in our current study that the IMGs experienced fewer clinical cross-cultural challenges may be that many of the IMGs training in psychiatry had lived in Norway for several years and might have known the dominant Norwegian cultural norms well. The IMGs may also have been more experienced as practicing physicians before emigration. In addition, IMGs may be more aware and conscious of the actual cultural diversity of which they are a part (Kirmayer et al., 2018). Their experience of being immigrants themselves may have promoted awareness and better understanding of immigrant or ethnic minority patients. Ethnic Norwegian doctors are not exposed to other cultures to the same degree, and this may have limited their cultural awareness and skills.

Yet another explanation might be that IMGs might deny or minimize cross-cultural challenges out of a fear that they might be regarded as not qualified or competent enough in their work in the host country.

For their part, native-born Norwegian doctors most often lack knowledge about the cultural background of their foreign patients. They might perceive more difficulties in treating them, partly because of fewer experiences with cross-cultural consultations. Feelings of incompetence might lead to withdrawal from clinical encounters with patients from a foreign culture, thus compounding the problem (Leseth, 2015). However, a high frequency of cross-cultural consultations was a significant predictor of PCC, but only before adjusting for other variables, indicating that this explanation may play a less important role.

We do not know whether the difference in the level of PCC between the two groups was indeed real. It should be noted that self-reported clinical competency is not particularly valid. A previous Norwegian study comparing self-reports and observers’ ratings of clinical communication skills among young physicians demonstrated a lack of concordance between the physicians’ own assessment and observers’ assessments (Gude et al., 2017). Physicians who reported that they performed very well, were observed to communicate more poorly than those who rated their own performance lower. Therefore, further research, preferably with a mixed-method sequential design to study both qualitative and quantitative data is needed to clarify this issue. Such a study could also very well include personality trait assessment.

### Factors associated with perceived cross-cultural clinical challenges

The only independent variable that significantly predicted a high level of PCC in both groups was work–home interface conflict. Difficulties in balancing work and home life have previously been shown as the only type of work-related stress to increase during the first years of specialty training of Norwegian doctors (Røvik et al., 2007). High work–home conflict has also been found among Canadian foreign residents in psychiatry and surgery (Sockalingam, Hawa et al., 2014; Sockalingam, Khan et al., 2014). Further, studies have shown that work–home conflict was an independent predictor of burnout (or emotional exhaustion) in physicians (Hertzberg et al., 2015; Langballe et al., 2011; Linzer et al., 2001). To our knowledge, the current study is the first to show that work–home interference also impacts doctors’ perceptions of difficulties in psychiatric clinical work with patients from a different culture and, as such, may interfere with their performance and functioning. This should be further studied. It is noteworthy that the work–home interference effect on PCC was adjusted for neuroticism, which implies that this was an independent effect.

### Limitations and strengths of the study

To make our study operational, we used a simplification of the concept of culture and cultural diversity. It should be noted that the dichotomization into IMGs and native-born Norwegian doctors does not represent the actual ethno-cultural heterogeneity of doctors and does not do justice to the fact that the population in Norway includes national minorities like the indigenous Sámi people in addition to second- and third-plus generation immigrants. A broader study with more differentiated categories, taking doctors’ ethnicity, social background, and country of origin into account, would have improved the validity. However, this was not feasible with the relatively small number of respondents included in this study. Further, one might argue that the number of years living in Norway might have had an impact on PCC among the IMGs. We tested this in an additional analysis, but the association proved to be non-significant in our relatively small sample.

Our investigation was limited to doctors’ perceptions of their most important clinical challenges when assessing patients from another culture. It did not include important topics such as language problems and communication skills in cross-cultural encounters. Problems were seen from the doctor's perspective, not from the patient's perspective. Hence, focusing on “cultural safety” (creating a safe environment for minority patients) and on power inequality in the doctor–patient relationship were outside the scope of the study. Mental health care in a cross-cultural context is indeed complex and requires more nuance as to the concept of culture and cultural diversity than provided by the current study.

A major limitation of this study is that our findings are based on the participants’ own judgment. As to perception of skills and performance, observation-based data could have given additional information. Hence, self-reports should be complemented by observed performance as the doctors may have either underestimated or overestimated their own difficulties (Tyssen, 2018). Still, perceptions among the trainees are certainly of great value in identifying their perceived challenges. Further, the questionnaires were answered at the end of a lecture in a mandatory course, which might have limited the time for reflection.

Another limitation is that there may be other important work-related or individual factors impacting PCC that we have not measured. For instance, other studies have shown that insufficient supervision from senior colleagues, poor learning climate, legal and ethical issues related to coercive treatment, time-consuming paperwork, and threats and violence from patients are quite stressful factors for young doctors working in psychiatry (Chan et al., 2019; Nøland et al., 2021; Røvik et al., 2007).

Further, we did not know the trainees’ professional orientation other than the fact that all trainees in Norway receive mandatory psychodynamic oriented psychotherapy supervision. A relationally oriented and patient-centered trainee might experience cross-cultural challenges differently from a trainee who identifies him/herself with a more authoritarian role. Such individual factors were not included in our study. A more extensive study, including more possible predictors of PCC, is recommended for future research. A mixed-method approach with more qualitative data could also give us more relevant information, e.g., about the contents of tools that are needed.

An obvious strength of the current study is the representativeness of the sample due to an extraordinary high response rate (93%), as well as the development of a new and reliable inventory to measure what challenges doctors in psychiatry experience when assessing and treating patients from different cultures. Our instrument measured cross-cultural challenges closely linked to clinical work and patient care in psychiatry.

## Implications

This study explored doctors’ perceptions of challenges in cross-cultural psychiatric work and, hence, may be of relevance to clinicians working in this field. Our findings suggest that mentoring and training programs in psychiatry should focus more on specific clinical challenges that trainees experience when they meet patients from different cultures. IMGs and native doctors might experience cross-cultural encounters with patients differently and can possibly benefit from mutual learning. Not least, this applies to the native trainees in psychiatry since they experienced cross-cultural patient work as being more demanding.

Our study further suggests that work-related factors, such as work–home conflict may impact doctors’ perceptions of difficulties in cross-cultural clinical work. Hence, mentoring and training programs should also include ways in which doctors balance home life and work, and how work–home interference may impact their perception of their own cross-cultural clinical performance.

## Conclusion

Native-born Norwegian doctors reported higher levels of PCC than did IMGs. Independent factors associated with higher PCC included native-born Norwegian doctor and experiencing high levels of work–home conflict. Both native doctors and IMGs rated “assessing psychosis,” “assessing suicide risk,” and “lacking tools in cross-cultural consultations” as the most demanding challenges in cross-cultural consultations. Assessment of psychosis and suicide risk are skills that are highly dependent on language skills as well as cultural understanding and cultural sensitivity.

## Supplemental Material

sj-docx-1-tps-10.1177_13634615241296297 - Supplemental material for Perceived clinical challenges when treating patients from different cultures: A study among psychiatry trainees in Norway Supplemental material, sj-docx-1-tps-10.1177_13634615241296297 for Perceived clinical challenges when treating patients from different cultures: A study among psychiatry trainees in Norway by Morten Sandbu, Anne Cecilie Javo, Suraj Bahadur Thapa, Karin Isaksson Rø, Valjbona Preljevic and Reidar Tyssen in Transcultural Psychiatry

## References

[bibr1-13634615241296297] Aggarwal N. K. Jarvis G. E. Gómez-Carrillo A. Kirmayer L. J. Lewis-Fernández R. (2020). The cultural formulation interview since DSM-5: Prospects for training, research, and clinical practice. Transcultural Psychiatry, 57, 496–514. 10.1177/1363461520940481 32838655

[bibr2-13634615241296297] American Psychiatric Association. (2013). Diagnostic and statistical manual of mental disorders: DSM-5 (5th ed.). American Psychiatric Association.

[bibr3-13634615241296297] Bäärnhielm S. Edlund A.-S. Ioannou M. Dahlin M. (2014). Approaching the vulnerability of refugees: Evaluation of cross-cultural psychiatric training of staff in mental health care and refugee reception in Sweden. BMC Medical Education, 14, 207–217. 10.1186/1472-6920-14-207 25262446 PMC4189165

[bibr4-13634615241296297] Bachmann S. (2018). Epidemiology of suicide and the psychiatric perspective. International Journal of Environmental Research & Public Health, 15, 1425. 10.3390/ijerph15071425 29986446 PMC6068947

[bibr5-13634615241296297] Belfrage A. Grotmol K. S. Tyssen R. Moum T. Lien L. (2018). Factors associated with low vs increased perceived mastery of clinical work over ten years of practice: A prospective study of Norwegian doctors. BMC Medical Education, 18, 116. 10.1186/s12909-018-1236-9 29843695 PMC5975549

[bibr6-13634615241296297] Bowling A. (2023). Research methods in health: investigating health and health services, chapters 13 and 15 (5th ed.). Open University Press.

[bibr7-13634615241296297] Chan M. K. Chew Q. H. Sim K. (2019). Burnout and associated factors in psychiatry residents: A systematic review. International Journal of Medical Education, 10, 149–160. 10.5116/ijme.5d21.b621. PMID: 31381505 31381505 PMC6766386

[bibr8-13634615241296297] Cruzeiro Szortyka A. L. S. Faria N. M. X. Carvalho M. P. Feijó F. R. Meucci R. D. Flesch B. D. Fiori N. S. Fassa A. G. (2021). Suicidality among south Brazilian tobacco growers. Neurotoxicology (Park Forest South), 86, 52–58. 10.1016/j.neur.2021.06.005 34214458

[bibr9-13634615241296297] Depue R. A. Monroe S. M. (1986). Conceptualization and measurement of human disorder in life stress research: The problem of chronic disturbance. Psychological Bulletin, 99, 36–51. 10.1037/0033-2909.99.1.36 3704034

[bibr10-13634615241296297] Ekblad S. Kastrup M. C. (2013). Current research in transcultural psychiatry in the Nordic countries. Transcultural Psychiatry, 50, 841–857. 10.1177/1363461513511181 24301661

[bibr11-13634615241296297] Fung H. W. Chan C. Ross C. A. (2020). Clinical correlates of hearing voices among people seeking interventions for dissociation: A cross-cultural investigation. Psychosis, 12, 328–338. 10.1080/17522439.2020.1773910

[bibr12-13634615241296297] Gude T. Finset A. Anvik T. Bærheim A. Fasmer O. B. Grimstad H. Vaglum P. (2017). Do medical students and young physicians assess reliably their self-efficacy regarding communication skills? A prospective study from end of medical school until end of internship. BMC Medical Education, 17, 107–114. 10.1186/s12909-017-0943-y 28666440 PMC5493874

[bibr13-13634615241296297] Hall P. Keely E. Dojeiji S. Byszewski A. Marks M. (2004). Communication skills, cultural challenges and individual support: Challenges of international medical graduates in a Canadian healthcare environment. Medical Teacher, 26, 120–125. 10.1080/01421590310001653982 15203520

[bibr14-13634615241296297] Hertzberg T. K. Rø K. I. Vaglum P. J. Moum T. Røvik J. O. Gude T. Ekeberg Ø Tyssen R. (2015). Work-home interface stress: An important predictor of emotional exhaustion 15 years into a medical career. Industrial Health, 54(2), 139–148. 10.2486/indhealth.2015-0134. Epub. Nov 3 26538002 PMC4821897

[bibr15-13634615241296297] Islam Z. Rabiee F. Singh S. P. (2015). Black and minority ethnic Groups’ perception and experience of early intervention in psychosis services in the United Kingdom. Journal of Cross-Cultural Psychology, 46, 737–753. 10.1177/0022022115575737

[bibr16-13634615241296297] Kirmayer L. J. Aggarwal N. K. Lewis-Fernández R. Hinton L. Hinton D. E. (2016). American Psychiatric A. DSM-5® handbook on the cultural formulation interview. American Psychiatric Publishing Inc.

[bibr17-13634615241296297] Kirmayer L. J. Sockalingam S. Fung K. P. Fleisher W. P. Adeponle A. Bhat V. Munshi A. Ganesan S. (2018). International medical graduates in psychiatry: Cultural issues in training and continuing professional development. Canadian Journal of Psychiatry, 63, 258–280. 10.1177/0706743717752913 29630854 PMC5894917

[bibr18-13634615241296297] Lai R. Plakiotis C. (2020). Stress and wellbeing of psychiatry trainees: A literature review. In P. Vlamos (ed.) GeNeDis 2018. Advances in Experimental Medicine and Biology (vol 1195, pp. 117–126). Springer, Cham. 10.1007/978-3-030-32633-3_16 32468466

[bibr19-13634615241296297] Langballe E. M. Innstrand S. T. Aasland O. G. Falkum E. (2011). The predictive value of individual factors, work-related factors, and work-home interaction on burnout in female and male physicians: A longitudinal study. Stress and Health, 27, 73–87. 10.1002/smi.1321

[bibr20-13634615241296297] Laroi F. Luhrmann T. M. Bell V. Christian W. A. Deshpande S. Fernyhough C. Janis J. Angela W. (2018). Culture and hallucinations: Overview and future directions. Schizophrenia Bulletin (2014), 40, S213–S220. 10.1093/schbul/sbu012 PMC4141319 24936082

[bibr21-13634615241296297] Launer J. (2018). Doctors as migrants. Postgraduate Medical Journal, 94, 543–544. 10.1136/postgradmedj-2018-136037 30381387

[bibr22-13634615241296297] Lehti A. Hammarström A. Mattsson B. (2009). Recognition of depression in people of different cultures: A qualitative study. BMC Family Practice, 10, 53–53. 10.1186/1471-2296-10-53 19635159 PMC2723088

[bibr23-13634615241296297] Leseth A. B. (2015). What is culturally informed psychiatry? Cultural understanding and withdrawal in the clinical encounter. BJPsych Bulletin, 39(4), 187–190. 10.1192/pb.bp.114.047936 26755952 PMC4706137

[bibr24-13634615241296297] Lew B. Kõlves K. Zhang J. Zhizhong W. Koenig H. G. Yip P. S. F. Abu Talib M. Osman A. Siau C. S. Chan C. M. H. (2021). Religious affiliation and suicidality among college students in China: A cross-sectional study across six provinces. PloS One, 16(5), e0251698. 10.1371/journal.pone.0251698 PMC8133455 34010317

[bibr25-13634615241296297] Lewis-Fernández R. Aggarwal N. K. Kirmayer L. J. (2020). The cultural formulation interview: Progress to date and future directions. Transcultural Psychiatry, 57, 487–496. 10.1177/1363461520938273 32838656

[bibr26-13634615241296297] Linzer M. Visser M. R. M. Oort F. J. Smets E. M. A. McMurray J. E. de Haes H. C. J. M. (2001). Predicting and preventing physician burnout: Results from the United States and The Netherlands. American Journal of Medicine, 111, 170–175. 10.1016/S0002-9343(01)00814-2 11498074

[bibr27-13634615241296297] Lizardi D. Gearing R. E. (2010). Religion and suicide: Buddhism, native American and African religions, atheism, and agnosticism. Journal of Religion and Health, 49, 377–384. 10.1007/s10943-009-9248-8 19347586

[bibr28-13634615241296297] Mahajan J. Stark P. (2007). Barriers to education of overseas doctors in paediatrics: A qualitative study in south Yorkshire. Archives of Disease in Childhood, 92, 219–223. 10.1136/adc.2006.098939 16935914 PMC2083415

[bibr29-13634615241296297] Memon A. Taylor K. Mohebati L. M. Sundin J. Cooper M. Scanlon T. de Visser R. (2016a). Perceived barriers to accessing mental health services among black and minority ethnic (BME) communities: A qualitative study in southeast England. BMJ Open, 6, e012337–e012346. 10.1136/bmjopen-2016-012337 PMC5128839 27852712

[bibr30-13634615241296297] Memon A. P. Taylor K. M. Mohebati L. M. P. Sundin J. P. Cooper M. P. Scanlon T. M. de Visser R. (2016b). Perceived barriers to accessing mental health services among black and minority ethnic communities: A qualitative study in southeast England. The Lancet (British Edition), 388, S76–S76. 10.1016/S0140-6736(16)32312-1 PMC5128839 27852712

[bibr31-13634615241296297] Michalski K. Farhan N. Motschall E. Vach W. Boeker M. (2017). Dealing with foreign cultural paradigms: A systematic review on intercultural challenges of international medical graduates. PLoS One, 12(7), e0181330. 10.1371/journal.pone.0181330 PMC5513557 28715467

[bibr32-13634615241296297] Mills S. Xiao A. Q. Wolitzky-Taylor K. Lim R. Lu F. G. (2017). Training on the DSM-5 CFI improves cultural competence in general psychiatry residents: A pilot study. Transcultural Psychiatry, 54, 179–191. 10.1177/1363461517700812 28358239

[bibr33-13634615241296297] Nøland S. T. Taipale H. Mahmood J. I. Tyssen R. (2021). Analysis of career stage, gender, and personality and workplace violence in a 20-year nationwide cohort of physicians in Norway. R JAMA Netw Open, 4, e2114749. 10.1001/jamanetworkopen.2021.14749 PMC8239948 34181010

[bibr34-13634615241296297] Ramírez Stege A. M. Yarris K. E. (2017). Culture in la clínica: Evaluating the utility of the cultural formulation interview (CFI) in a Mexican outpatient setting. Transcultural Psychiatry, 54, 466–487. 10.1177/1363461517716051 28691591

[bibr35-13634615241296297] Rosmarin D. H. Bigda-Peyton J. S. Öngur D. Pargament K. I. Björgvinsson T. (2013). Religious coping among psychotic patients: Relevance to suicidality and treatment outcomes. Psychiatry Research, 210, 182–187. 10.1016/j.psychres.2013.03.023 23684053

[bibr36-13634615241296297] Rosso M. S. Bäärnhielm S. (2012). Use of the cultural formulation in Stockholm: A qualitative study of mental illness experience among migrants. Transcultural Psychiatry, 49, 283–301. 10.1177/1363461512442344 22508638

[bibr37-13634615241296297] Rotstein S. Jenkins K. (2017). Career satisfaction and work stressors in psychiatrists and psychiatry trainees in Australia and New Zealand. Australasian Psychiatry, 25, 172–174. 10.1177/1039856216684715 28068829

[bibr38-13634615241296297] Røvik J. O. Tyssen R. Hem E. Gude T. Ekeberg Ø Moum T. Vaglum P. (2007). Job stress in young physicians with an emphasis on the work-home interface: A nine-year, nationwide and longitudinal study of its course and predictors. Industrial Health, 45, 662–671. 10.2486/indhealth.45.662 18057809

[bibr39-13634615241296297] Sandbu M. Kamps A. Preljevic V. Javo C. (2015). Foreign doctors in Norwegian psychiatry – is there a need for a mentoring scheme? Tidsskrift for den Norske Laegeforening, 135, 1133–1137. 10.4045/tidsskr.14.0764 26130546

[bibr40-13634615241296297] Şar V. (2022). Dissociation Across Cultures: A Transdiagnostic Guide for Clinical Assessment and Management. Alpha Psychiatry. 23(3), 95–103. 10.5152/alphapsychiatry.2022.21556 PMC9590661 36425778

[bibr41-13634615241296297] Simon G. E. Johnson E. Lawrence J. M. Rossom R. C. Ahmedani B. Lynch F. L. Beck A. Waitzfelder B. Ziebell R. Penfold R. B. Shortreed S. M. (2018). Predicting suicide attempts and suicide deaths following outpatient visits using electronic health records. American Journal of Psychiatry, 175, 951–960. 10.1176/appi.ajp.2018.17101167 29792051 PMC6167136

[bibr42-13634615241296297] Sockalingam S. Hawa R. Al-Battran M. Abbey S. E. Zaretsky A. (2014). Preparing international medical graduates for psychiatry residency: A multi-site needs assessment. Academic Psychiatry, 36, 277–281. 10.1176/appi.ap.09110219 22851023

[bibr43-13634615241296297] Sockalingam S. Khan A. Tan A. Hawa R. Abbey S. Jackson T. Zaretsky A. Okrainec A. (2014). A framework for understanding international medical graduate challenges during transition into fellowship programs. Teaching and Learning in Medicine, 26, 401–408. 10.1080/10401334.2014.945393 25318037

[bibr44-13634615241296297] Stack S. Kposowa A. J. (2008). The association of suicide rates with individual-level suicide attitudes: A cross-national analysis. Social Science Quarterly, 89, 39–59. 10.1111/j.1540-6237.2008.00520.x

[bibr45-13634615241296297] Strand M. Baarnhielm S. (2022). Could the DSM-5 cultural interview hold therapeutic potential? Suggestions for further exploration and adaptation within a framework of therapeutic assessment? Culture, Medicine and Psychiatry, 46, 846–863. 10.1007/s11013-021-09761-2 34881417 PMC9596502

[bibr46-13634615241296297] Sturesson L. Öhlander M. Nilsson G. H. Palmgren P. J. Stenfors T. (2019). Migrant physicians’ entrance and advancement in the Swedish medical labour market: A cross-sectional study. Human Resources for Health, 17, 71–86. 10.1186/s12960-019-0414-0 31615515 PMC6794744

[bibr47-13634615241296297] Sutherland V. J. Cooper C. L. (1992). Job stress, satisfaction, and mental health among general practitioners before and after introduction of new contract. BMJ, 304, 1545–1548. 10.1136/bmj.304.6841.1545 1628056 PMC1882446

[bibr48-13634615241296297] Torgersen S. (1980). Hereditary-environmental differentiation of general neurotic, obsessive, and impulsive hysterical personality traits. Acta Geneticae Medicae et Gemellologiae, 29, 193–207. 10.1017/S0001566000007935 7196669

[bibr49-13634615241296297] Turecki G. P. Brent D. A. P. (2015). Suicide and suicidal behaviour. Lancet, 387, 1227–1239. 10.1016/S0140-6736(15)00234-2 26385066 PMC5319859

[bibr50-13634615241296297] Tyssen R. (2018). What is the level of burnout that impairs functioning? Journal of Internal Medicine, 283, 594–596. 10.1111/joim.12769 29878500

[bibr51-13634615241296297] Tyssen R. Vaglum P. Grønvold N. T. Ekeberg Ø (2000). The impact of job stress and working conditions on mental health problems among junior house officers. A nationwide Norwegian prospective cohort study. Medical Education, 34, 374–384. 10.1046/j.1365-2923.2000.00540.x 10760123

