# Supplementary material for: Perceived clinical challenges when treating patients from different cultures: A study among psychiatry trainees in Norway
Source: Transcult Psychiatry. 2024 Dec 24;62(2):181–91. doi: 10.1177/13634615241296297 (PMC12130610; doi:10.1177/13634615241296297)
Supplement: sj-docx-1-tps-10.1177_13634615241296297 - Supplemental material for Perceived clinical challenges when treating patients from different cultures: A study among psychiatry trainees in Norway [file sj-docx-1-tps-10.1177_13634615241296297.docx]

**Appendix 1.**

The following statements were scored on a 7-point Likert scale, from 1 (incorrect) to 7 (fits well):

1. Contact frequency is not influenced by the patient having the same or a different mother tongue or cultural background than my own
2. According to my experience, the current psychiatric service offered is relevant and sufficient for immigrant patients
3. I send a copy of the discharge note to patients whose linguistic/cultural background is significantly different from my own
4. I find it difficult to create a treatment plan if the patient has a linguistic/cultural background that is significantly different from my own
5. I find it demanding to assess suicide risk among patients whose linguistic/cultural background is significantly different from my own
6. I find it demanding to assess violence risk among patients whose linguistic/cultural background is significantly different from my own
7. I lack tools when I meet patients whose cultural background is different from my own
8. I have experienced linguistic differences as stimulating and valuable when meeting patients
9. As a foreign doctor, I have experienced advantages when I treat Norwegian patients
10. As a foreign doctor, I have experienced advantages when I treat foreign patients
11. As a native Norwegian doctor, I have experienced advantages when I treat native Norwegian patients
12. As a native Norwegian doctor, I have experienced advantages when I treat immigrant patients
13. As a foreign doctor, I have experienced challenges when treating native Norwegian patients
14. As a foreign doctor, I have experienced challenges when treating foreign patients
15. As a native Norwegian doctor, I have experienced challenges when treating native Norwegian patients
16. As a native Norwegian doctor, I have experienced challenges when treating immigrant patients
17. If the patient’s linguistic or cultural background is different from my own, I have occasionally used the patient herself/himself as an ambassador for her/his own culture
18. I experience assessing psychosis as being more demanding with patients whose linguistic or cultural background is different from my own
19. I experience that assessing whether a religious practice is exaggerated or “morbid" as being more demanding among patients whose religious background is different from my own
20. I experience that assessing treatment outcomes as being more demanding with patients whose linguistic or cultural background is different from my own
21. I find it more difficult to include patients whose linguistic or cultural background is different from my own in shared decision making
22. I have sufficient clinical skills to treat patients with psychiatric diseases who have a different cultural background than me
23. I have sufficient cultural understanding to treat patients with psychiatric diseases whose cultural background is different from my own
24. I have got sufficient communication skills to treat patients with psychiatric diseases who have a different cultural background than me
